# Pulmonary thromboembolism and alveolar hemorrhage as initial manifestations of systemic lupus erythematosus

**DOI:** 10.1177/09612033211066481

**Published:** 2022-01-18

**Authors:** O. Jiménez-Zarazúa, L. N. Vélez-Ramírez, C. A. Ramírez-Casillas, J. D. Mondragón

**Affiliations:** 1 Hospital General Regional IMSS No. 21, 42581 Department of Internal Medicine, León, Guanajuato, Mexico; 2 Department of Medicine and Nutrition, 10173 Universidad de Guanajuato, Guanajuato, Mexico; 3 Hospital General de León, Department of Radiology, León, Guanajuato, Mexico; 4 University of Groningen, University Medical Center Groningen, Department of Neurology, The Netherlands; 5 University of Groningen, University Medical Center Groningen, Alzheimer Center Groningen, The Netherlands

**Keywords:** alveolar hemorrhage, systemic lupus erythematosus, pulmonary thromboembolism

## Abstract

Systemic lupus erythematosus (SLE) is an autoimmune disease that affects multiple organs. SLE can affect the lung, the pulmonary vasculature, and the pleura. A 38-year-old female with limb pain and ecchymosis who later developed pulmonary thromboembolism and alveolar hemorrhage is presented here. Clinical, imaging, laboratory, and histopathological evidence is presented. The patient met the European League Against Rheumatism (EULAR) and the American College of Rheumatology (ACR) criteria for SLE. Furthermore, the patient had a Systemic Lupus Erythematosus Disease Activity Index 2000 (SLEDAI-2K) score of 35; thus, indicating severe disease. This case is an example of concomitant venous and arterial lung complications in an SLE patient.

## Introduction

Systemic lupus erythematosus (SLE) is an autoimmune disease that affects multiple organs. It is characterized by the production of different antibodies (e.g., anti-nuclear antibodies, ANA, and/or anti-dsDNA antibodies, anti-Ro/La, anti-RNP, and anti-Sm antibodies),^1^ as well as having different clinical presentations (e.g., glomerulonephritis, cytopenia, arthritis, and photosensitive rash).^2^ The prevalence of SLE has been estimated to be 30–50 per 100,000, which is equivalent to 500,000 patients in Europe and 250,000 in the United States of North America.^3^ The respiratory system can be involved in up to 50–70% of SLE patients and be the initial manifestation in 4–5% of the cases.^4^ SLE can affect various structures of the respiratory system: 1) the lung (i.e., interstitial pneumonia, bronchiolitis obliterans with pneumonia, acute lupus pneumonitis); 2) the vasculature (i.e., pulmonary hypertension, alveolar hemorrhage, reversible acute hypoxemia, and antiphospholipid syndrome); and 3) the pleura.^5,6^ Pleurisy can be present in up to 60% of SLE, while chronic interstitial pneumonia is 3–13%, and alveolar hemorrhage is less than 2%.^7^ The risk of complications and mortality depend on the type and extent of respiratory involvement and the presence of comorbidities.^4^

In the case presented below, a patient with SLE debuted with a clinical pulmonary involvement; ultimately, suffering from pulmonary thromboembolism and alveolar hemorrhage. In the discussion, we provide the reader with the relevant literature to put this case into clinical context. This case report should provide the clinician with an example of a rare presentation of SLE involving concomitant venous and arterial pulmonary complications.

### Case presentation

A 38-year-old female arrived at the Emergency Department with oppressive pain in the right pelvic limb with a 6/10 intensity on the analogous visual scale, with increased volume and ecchymosis at the posterior tibial region. The patient did not allude to any exacerbating or mitigating causes. The patient’s family history included a mother with type 2 diabetes and a father with arterial hypertension (AH); other relevant aspects of family history were questioned and denied. Among the patient’s personal history, tobacco consumption is reported for more than 10 years at a rate of three cigarettes per day (i.e., 30 pack-years), but currently, the patient refers to be a non-smoker. The patient denied a history of allergies, blood transfusions, recent travel, tattoos, and piercings. The patient had a 2-year history of AH, treated with calcium channel blockers, angiotensin II receptor antagonists, and beta-blockers. The patient also referred a personal history of deep vein thrombosis 2-years prior treated with anticoagulant (i.e., acenocoumarin, weaned off for over 12 months). The patient had no history of lung disease, asthma during childhood, and chronic or degenerative disease. Obstetric history includes six gestations, one miscarriage, one cesarean delivery, and four normal deliveries.

Upon admission, the patient had the following vital signs: blood pressure 110/80 mmHg; heart rate 110 bpm; respiratory rate 20 rpm; oxygen saturation of 88% with noninvasive ventilation at a rate of 10 L/min; body temperature 37°C. We found a patient with a Glasgow coma score of 15 points, somnolent, without focal neurologic deficits nor meningeal signs, aware of her environment, with reference to place, time, and people. Upon inspection, abdominal dissociation and accessory muscles use were noted. The thorax had decreased expansion without vibrations or fremitus during palpation. No asymmetries or abnormal findings in tone intensity, pitch, duration, and quality through direct percussion. Precordial auscultation reveals tachycardia 110 bpm, heart sounds of good intensity without extra heart sounds. The lower right extremity with edema, hyperemia, increased local temperature with ecchymosis, and fovea ++/+++. The Homan’s sign and Pratt test were positive for deep vein thrombosis; Lowenberg’s and Bancroft’s signs were not present. Wells score of 4 (i.e., 1), Calf swelling >3 cm compared to the other leg, 2) localized tenderness along the deep venous system, 3) pitting edema, confined to symptomatic leg, and 4) previously documented DVT was reported for deep vein thrombosis (i.e., high risk).

**Figure 1.**
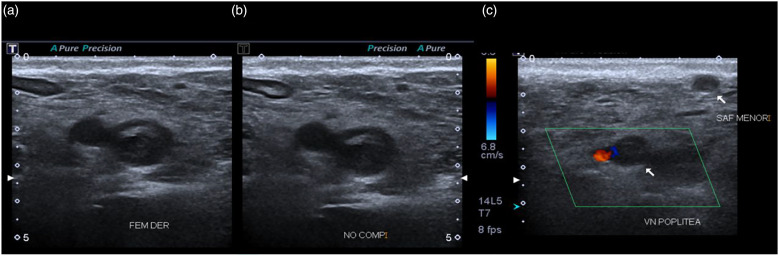
Venous ultrasonography showing thrombi. **(A)** Grayscale ultrasound at the level of the right common femoral artery, where an echogenic image can be seen in its interior compatible with a thrombus. **(B)** The lack of compression of said vessel is demonstrated. **(C)** Popliteal vein color Doppler ultrasound showing a hypoechoic image that occupies the interior of the vessel without evidence of a spontaneous flow signal, the interior of the lesser saphenous vein with the presence of a thrombus inside can also be observed.

**Figure 2.**
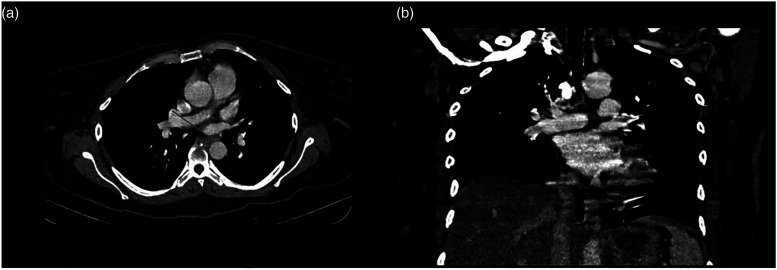
Computed tomography angiography of pulmonary vasculature showing embolism. Computed tomography angiography of the pulmonary vasculature. **(A)** Axial cut and **(B)** coronal reconstructions of the thorax in a contrasted phase showing the main branch of the right pulmonary artery. A filling defect with angulated edges, mainly occupying the ascending branch and a small portion of the descending one is observed (i.e., pulmonary embolism).

Laboratory results upon admission are presented in Table 1. Toxicology laboratories were negative for hepatitis B and hepatitis C virus and HIV (Table 2). Chest X-ray, posterior**–**anterior projection, shows increased cardiac silhouette without any other radiographic alteration. Lower extremity Doppler ultrasound was performed reporting an echogenic image compatible with a thrombus localized at the right common femoral artery; furthermore, the popliteal vein and the small saphenous vein with the presence of thrombi (Figures 1(a)–(c)). A computed tomography angiography (CTA) was performed and reported the right pulmonary artery with an image compatible with a pulmonary embolism (PE; Figures 2(a)–(b)).

**Table 1.** Laboratory test results upon admission.

Full blood count							Liver enzymes				
Hemoglobin at admission				9.2 g/dL			Total bilirubin			1.50 mg/dL	
Hematocrit				27.8%			Indirect bilirubin			0.20 mg/dL	
Erythrocyte count				3.3 µL			Direct bilirubin			1.30 mg/dL	
Platelet count				169,000 µL			Total protein			5.70 g/dL	
Mean corpuscular volume				84.6 fL			Albumin			2.10 g/dL	
Mean corpuscular hemoglobin				28 g/dL			Globulins			3.60 g/dL	
Leukocyte count				40,000 µL			Alanine aminotransferase			67 U/L	
Neutrophils				76%			Aspartate aminotransferase			145 U/L	
Lymphocytes				18%			Lactate dehydrogenase			456 U/L	
Monocytes				5%			Urinalysis				
Eosinophils				0%			Appearance			Cloudy	
Basophils				1%			pH			6	
Blood chemistry				Specific gravity			1.015				
Glucose				104 mg/dL			Proteins			30 mg/dL	
Albumin				2.1 gr/dL			Ketones, glucose, and nitrite			Negative	
Urea nitrogen				92 mg/dL			Leukocytes			75/High power field	
Blood urea nitrogen				42 mg/dL			Erythrocytes			Uncountable	
Uric acid				10 mg/dL			Bacteria			Abundant	
Creatinine				3.9 mg/dL			Benzodiazepines			Negative	
Blood coagulation				Barbiturates			Negative				
Prothrombin time				13.5 sec			Cannabis			Negative	
Partial thromboplastin time				54 sec			Cocaine			Negative	
International normalized ratio				1.16			Methamphetamines			Negative	
Electrolytes				Opiates			Negative				
Sodium				134 mEq/dL			Thyroid function tests				
Potassium				6.2 mEq/dL			Serum thyroxine (T4)			9 μg/dL	
Chlorine				103 mEq/dL			Free thyroxine (fT4)			1 ng/dL	
Calcium				8.6 mg/dL			Serum triiodothyronine (T3)			0.9 ng/mL	
Phosphorus				4 mg/dL			T3 resin uptake (T3RU)			3 pg/mL	
Magnesium				2 mEq/dL			Serum thyrotropin (TSH)			2.5 μg/mL	
Muscle enzymes											
Creatine Kinase-MB (CKMB)				4 U/L			Creatine phosphokinase (CPK)			61 U/L	
Troponine I				0.226 ng/mL							
Blood gases											
pH	7.4	pCO_2_	20	pCO_3_	76	HCO_3_	14.2	Base excess	−9	SatO_2_	96%

**Table 2.** Follow-up laboratory test results.

Serum antibodies		Result	EULAR/ACR immunologic criteria for SLE
Cytoplasmic antineutrophil cytoplasmatic (cANCA)		Negative	
Perinuclear antineutrophil cytoplasmatic (pANCA)		Negative	
Cyclic citrullinated peptide	<2 U/mL	Negative	
Anti-SS-A (ro)	<1.0 U/mL	Negative	
Anti-SS-B (La)	<1.0 U/mL	Negative	
Anti-nuclear (ANA)	<1:10	Negative	
Anti-cardiolipin IgG	2.0 U/mL	Negative	0
Anti-cardiolipin IgM	2.0 U/mL	Negative	0
Beta-2 glycoprotein 1 IgG and IgM	<15.0 U/mL	Negative	0
Lupus anticoagulant	<1.0 U/mL	Negative	0
Complement C3	50 mg/dL	Decreased	2
Complement C4	10 mg/dL	Decreased	2
Anti-DNA double strain	45.9 IU/mL	Positive	6
Anti-Smith	2.9 U/mL	Positive	NA
Viral panel			
Hepatitis B virus		Negative	
Hepatitis C virus		Negative	
Human immunodeficiency virus		Negative	
Hematology			
Protein C		Normal	
Protein S		Normal	
Antithrombin III		Normal	
Factor V Leiden		Normal	
Homocysteine		Normal	

EULAR/ACR: European League Against Rheumatism and the American College of Rheumatology.

### Clinical evolution

After the diagnosis of PE was established, anticoagulant therapy was initiated with low molecular weight heparin. The patient presented hemodynamic instability 24 h after being admitted to the hospital. The patient had a blood pressure of 70/36 mmHg and a mean arterial pressure of 48 mmHg, as well as decreased awareness. Advanced airway management was initiated with endotracheal intubation and mechanical ventilation. A transthoracic echocardiogram (TTE) was performed at the request of the cardiology department, reporting middle segment right ventricular hypokinesia, pulmonary artery pressure of 32 mmHg, tricuspid annular plane systolic excursion 18 mm, right and left ventricle relationship 1.0, left ventricular ejection fraction of 35%, and cardiac output of 1.8 L/min.

**Figure 3.**
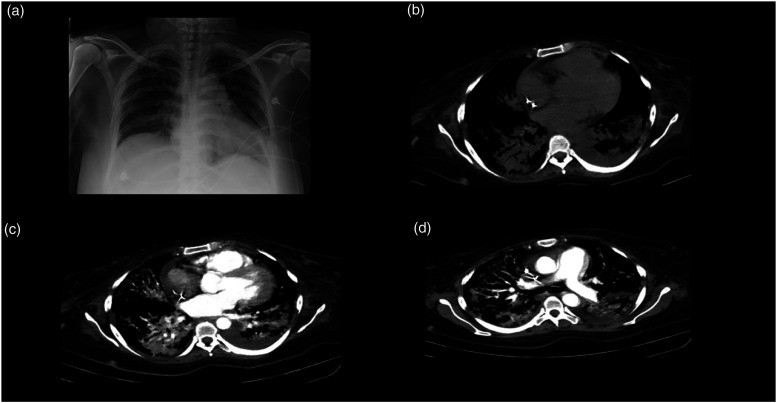
Follow-up chest X-ray and computed tomography. **(A)** Chest X-ray, posterior**–**anterior projection, showing increased radiopacity in both hemithorax. **(B–D)** Simple and contrasted axial computed tomography showing small areas of hypodensity with air bronchogram that converge in the posterior lobes, also seen bilateral pleural effusion with left predominance.

**Figure 4.**
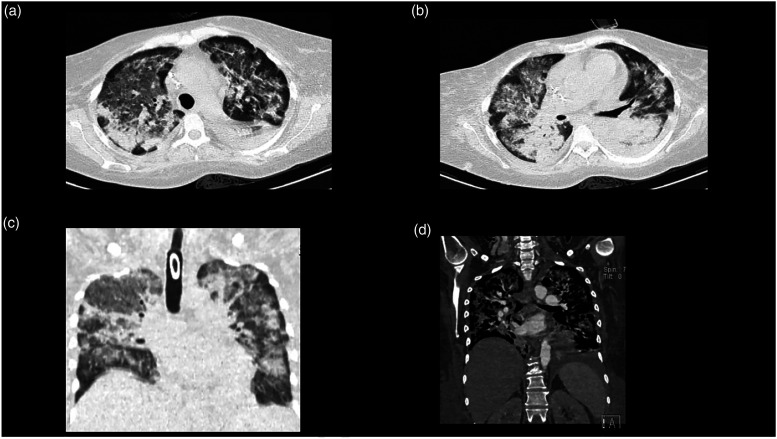
Follow-up lung computed tomography. Computed tomography (CT). **(A,B)** Axial sections with a lung window, increased bilateral density, consolidation, and air bronchogram are observed.) Coronal CT reconstruction with a lung window. **(C)** Generalized increase radiopacity with a small left basal consolidation area. **(D)** Coronal CT reconstruction with contrast, generalized increased density is seen in both lung parenchyma.

Vasopressors were initiated with norepinephrine (0.5 mcg/kg/min) and dobutamine (0.2 mcg/kg/min). Thrombolysis with alteplase 15 mg IV bolus was initiated, followed by a 50 mg IV 30-minute infusion and a 35 mg 60-minute infusion. After 72 h, the patient had anuria with a creatinine of 2.8 mg/dL, urea nitrogen of 146 mg/dL, and blood urea nitrogen of 68 mg/dL. Continuous renal replacement therapy (CRRT) was initiated with six sessions. On the ninth day of CRRT, the follow-up laboratory workup was creatinine of 1 mg/dL, urea nitrogen of 58 mg/dL, and blood urea nitrogen of 27 mg/dL; furthermore, the BUN/creatinine urinary output ratio was 1 mL/kg/hr. On day 11 of hospitalization, the patient had hemoptysis with a hemoglobin drop greater than 2 g/dL. A chest X-ray revealed increased radiodensity at both hemithorax (Figure 3(a)). Alveolar hemorrhage was suspected and two blood units were transfused. Table 3 reports the follow-up hemograms throughout the patient’s hospitalization. Blood cultures and bronchial secretion cultures were performed and reported negative. The patient did not accept to undergo bronchoscopy with bronchioalveolar lavage. A new chest CT scan was performed, revealing small hypodense regions with air bronchogram that merge at the posterior lobules (Figures 3(b), 4(a)(d)a–d4(a(d)), as well as bilateral pleural effusion with predominance in the left side (Figures 3(c)–(d)).

**Table 3.** Follow-up hemograms at different time points.

Complete blood count	Day 11th	Day 12th	Day 14th	Day 17th	Day 20th
Hemoglobin	6.3 g/dL	8.1 g/dL	7.1 g/dL	6.6 g/dL	8.2 g/dL
Hematocrit	19.6%	25.3%	22.0%	20.2%	24.7%
Erythrocyte count	2.1 µL	2.7 µL	2.4 µL	2.2 µL	2.8 µL
Platelet count	75,000 µL	82,900 µL	117,000 µL	130,000 µL	127,000 µL
Mean corpuscular volume	94.0 fL	93.2 fL	91.8 fL	90.4 fL	89.0 fL
Mean corpuscular hemoglobin	30.3 g/dL	29.9 g/dL	30.4 g/dL	29.7 g/dL	29.5 g/dL
Leukocyte count	9.0 µL	12.2 µL	12.1 µL	4.9 µL	5.7 µL
Neutrophils	80.7%	89.9%	94.5%	90.8%	85.4%
Lymphocytes	9.7%	5.2%	3.2%	6%	10.8%
Monocytes	5.5%	3.5%	1.1%	2.2%	3%
Eosinophils	3.7%	1.1%	1%	0.9%	0.6%
Basophils	0.4%	0.3%	0.2%	0.1%	0.2%

**Figure 5.**
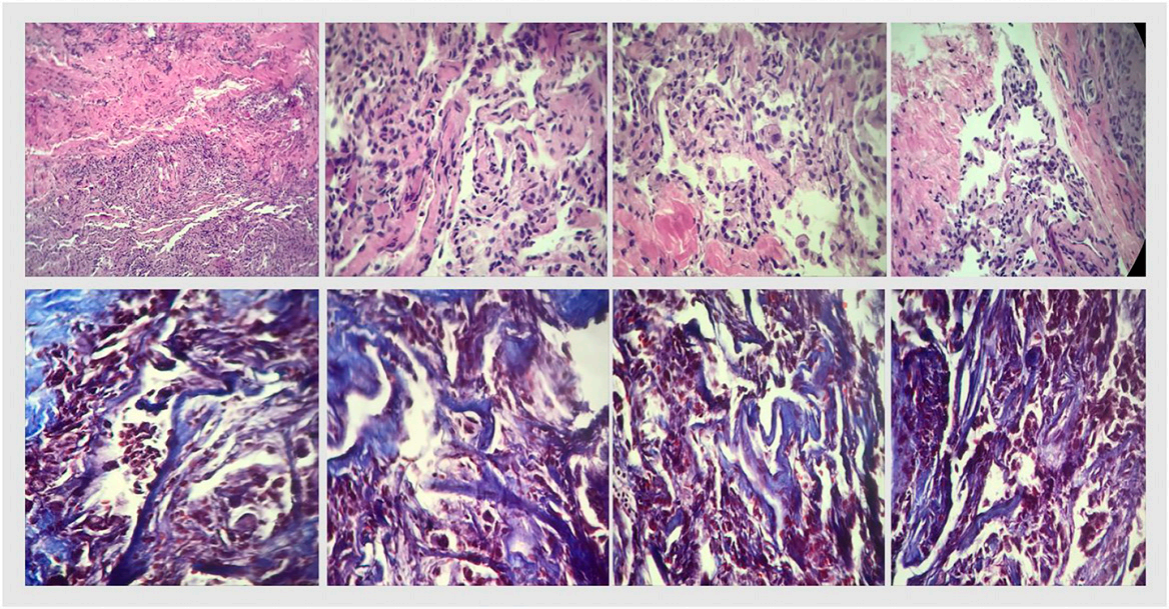
Pulmonary biopsy. Histopathological sections of the lung. Top row: hematoxylin and eosin staining. Bottom row: Masson’s trichrome staining. Thickening of the interstitial spaces (e.g., collagen seen in blue), chronic inflammation with lymphocytes and plasma cells can be observed.

In search of an autoimmune etiology, the following tests were requested: antineutrophil cytoplasmatic antibodies (ANCA), cytoplasmatic (cANCA), perinuclear (pANCA), cyclic citrullinated peptide, anti-SS-B (La), anti-SS-A (Ro), and anti-nuclear antibodies were all negative (Table 2). In search of SLE anti-cardiolipin IgM and IgG antibodies, Beta-2 glycoprotein 1 IgG and IgM antibodies, and lupus anticoagulant were negative; however, anti-double-stranded deoxyribonucleic acid anti-Smith antibodies were positive, as well as Complement C3 and C4 were decreased (Table 2). Thus, the diagnosis of SLE was integrated as per the European League Against Rheumatism (EULAR) and the American College of Rheumatology (ACR) criteria (Aringer et al., 2020); the patient had at least one clinical criteria (i.e., pleura effusion) and score ≥10 points (Table 2). After SLE diagnosis was integrated, treatment with three IV boluses of methylprednisolone 1 g was administered; followed by prednisone 0.5 mg/kg q24 h. Meanwhile, protein, S protein, antithrombin III, factor V Leiden, and homocysteine levels were reported normal. The patient continued with a torpid evolution secondary to active hemorrhage, which required transfusion of two more blood bags, raising hemoglobin levels from 6.3 g/dL to 8.2 g/dL (Table 3). After 20 days of hospitalization, the patient died after a cardiac arrest. A percutaneous pulmonary biopsy was performed *postmortem*, reporting septal and interstitial space thickening, lymphocyte and plasma B cell presence compatible with chronic interstitial inflammation (Figure 5).

## Discussion

We report the first recorded case of concomitant thromboembolism and alveolar hemorrhage associated with SLE. The patient met the SLE classification criteria^8^ and had a Systemic Lupus Erythematosus Disease Activity Index 2000 (SLEDAI-2K) score of 35; thus, indicating a severe disease.^1^ Deep vein thrombosis and pulmonary embolism occur in approximately 9% of SLE cases.^9^ Antiphospholipid antibodies (IgG and IgM) are associated with an increase in thromboembolic events up to 35–42%.^10^ However, the patient in the case presented above had negative antinuclear antibodies which is a very rare finding in SLE, as only approximately 6.2% of cases can be negative.^11^ Factors related to negative ANA antibodies are disease activity (i.e., systemic lupus erythematosus disease activity index score ≥10), lupus nephritis, and immunosuppressant use.^12^ The patient had an SLEDAI-2K score of 35; thus, a severe disease activity could be associated with the negative ANA antibodies laboratory result. Among other clinical manifestations associated with SLE are 1) pulmonary embolism, 2) lung infarction, 3) pulmonary hypertension, 4) pulmonary artery thrombosis, 5) pulmonary micro-thromboses, 6) acute respiratory distress syndrome, 7) diffuse alveolar hemorrhage, and 8) postpartum hemolytic uremic syndrome.^13^ In the subsequent paragraphs, we will put the case presented above into clinical context.

Alveolar hemorrhage was suspected in this patient due to the presence of hemoptysis, persistently decreased hemoglobin (>2 g/dL), as well as the presence of pulmonary infiltrates in the imaging studies.^14,15^ Early manifestations of SLE can be diverse and nonspecific (e.g., fatigue, fever, and malaise).^2,16^ Alveolar hemorrhage is a medical emergency that produces acute respiratory failure, requiring an early diagnosis, as well as aggressive treatment [17]. The radiological patterns suggestive of alveolar hemorrhage can be present 48–72 h after the clinical onset.^4^ SLE patients who present alveolar hemorrhage have approximately a 50% mortality rate.^18^ Advanced age, presence of massive hemoptysis, need for mechanical ventilation, previous plasmapheresis treatment, presence of thrombocytopenia, as well as infections have been associated with higher mortality risk.^4^ Alveolar hemorrhage secondary to SLE is more common in young women,^19^ as it was in this case. The following etiologies must be considered in the presence of alveolar hemorrhage: primary capillaritis (e.g., microscopic polyangiitis, ANCA-associated vasculitis, granulomatosis with polyangiitis, eosinophilic granulomatosis with polyangiitis, isolated pauci-immune pulmonary capillaritis, and Henoch–Schönlein purpura) and without capillaritis (e.g., SLE, scleroderma, primary anti-phospholipid syndrome, polymyositis, rheumatoid arthritis, mixed connective tissue disease, drug-induced vasculitis, IgA nephropathy, and anti-glomerular basement membrane antibody disease).^15,20,21^ Thrombocytopenia and low C3 levels are predictive factors for alveolar hemorrhage in SLE^14^; this case presented with reduced complement levels but not thrombocytopenia. Furthermore, although SLE patients have leucopenia, the patient in the case presented above had leukocytosis due to a septic process. Meanwhile, lupus nephritis has been linked with alveolar hemorrhage in SLE patients in 64–100%.^14^ Additionally, imaging studies can assist to diagnose alveolar hemorrhage in SLE patients; in chest X-ray, opacities can be seen in the central and basal airways, while in a chest CT, ground glass opacifications without significant interlobular septal thickening.^22^

Systemic lupus erythematosus has a three times greater risk of pulmonary embolism, a four times greater risk of deep vein thrombosis, and three times greater risk of venous thromboembolism than in the general population, a year after the diagnosis.^23^ Although the patient was not positive for the lupus anticoagulant, the patient had deep vein thrombosis. However, fifty percent of patients with SLE and lupus anticoagulants will present venous thrombosis within 20 years.^24^ Furthermore, lupus anticoagulants are a risk factor for the first event of venous thrombosis, as well as thrombi recurrence.^25^ Patients with SLE are at increased risk of venous thromboembolism events due to inflammation and/or the presence of antiphospholipid antibodies.^2^ In the case presented above, antiphospholipid antibodies were negative; however, SLE was further explored with a second antiphospholipid antibody determination.^25^ Other etiologies of thrombotic events, in this case, were excluded: protein C and S deficiency, antithrombin III and Factor V Leiden alterations, use of estrogens, among others.^26^

Management of alveolar hemorrhage in SLE has been previously successfully treated with corticosteroids and cyclophosphamide,^27^ as well as with pulse methylprednisolone, cyclophosphamide, and plasma exchanges.^28^ However, in the case presented above, the patient had a torpid evolution even after employing similar management. Among the treatment options available for alveolar hemorrhage in SLE are high corticosteroid doses (e.g., methylprednisolone), high immune-system suppressants (e.g., azathioprine, methotrexate, and mycophenolate), plasma exchanges, rituximab, belimumab.^3,16,21^ In the case presented above, the patient had alveolar hemorrhage 11 days after alteplase administration; while previous studies reported a similar complication after 4–5 days after the use of thrombolytic medication.^29,30^ Furthermore, a recent case series reported that alveolar hemorrhage secondary to thrombolytic administration was observed during the first 4–12 h posterior administration^31^; thus, the alveolar hemorrhage might have occurred irrespective of the thrombolysis. Among the multiple risk factors for alveolar hemorrhage that the patient did have were development thrombocytopenia and leukopenia, low C3 levels, high (ds)DNA levels, severe disease activity, and lupus nephritis.^4,14^ Finally, heart failure, in this case, could be associated with the pulmonary vasculature occlusion severity, which could have led to a right ventricular failure and a subsequent acute circulatory collapse. Furthermore, the patient had sepsis, which has been associated with cardiomyopathy (i.e., 20–50% of the cases), particularly, diastolic dysfunction, which can contribute to decreased cardiac output secondary to right ventricular failure, leading to biventricular failure, thus hemodynamic instability and death.^32, 33^

### Limitations

The patient refused to undergo a bronchial lavage to exclude an infectious alveolar hemorrhage origin. This is an important limitation as the patient had leukocytosis upon admission to the Internal Medicine department. Furthermore, the patient did not accept the management of his condition with rituximab or plasma exchanges, which could have positively impacted the patient’s prognosis.

## Conclusion

The case presented above is the first report in the literature that consists of concomitant thromboembolism and alveolar hemorrhage secondary to SLE. We present clinical, imaging, laboratory, and histopathological evidence. The patient met the European League Against Rheumatism (EULAR) and the American College of Rheumatology (ACR) criteria for SLE. Furthermore, the patient had a Systemic Lupus Erythematosus Disease Activity Index 2000 (SLEDAI-2K) score of 35; thus, indicating severe disease.

### Final Diagnosis

Thromboembolism and alveolar hemorrhage secondary to systemic lupus erythematosus disease.
